# The Short- and Long-Term Safety and Efficacy Profile of Subtotal Cholecystectomy: A Single-Centre, Long-Term, Follow-Up Study

**DOI:** 10.7759/cureus.44334

**Published:** 2023-08-29

**Authors:** Ahmed Salman Bodla, Muhammad Umair Rashid, Maleeha Hassan, Saad Rehman, George Kirby

**Affiliations:** 1 General Surgery, Shrewsbury and Telford Hospital NHS Trust, Shrewsbury, GBR

**Keywords:** gallstone disease (gsd), long-term follow-up, laparoscopic cholecystectomy, long-term outcomes, acute calculous cholecystitis, subtotal cholecystectomy

## Abstract

Background

Subtotal cholecystectomy (STC) has been reported as an effective method to remove the gallbladder if the hepatocystic triangle anatomy is unfavourable. However, the evidence regarding its long-term outcomes from the United Kingdom (UK) is lacking. This study aimed to assess its short and long-term outcomes with a minimum of one-year follow-up.

Methodology

We retrospectively analysed all elective and emergency STCs performed in a single UK NHS Trust between 2014 and 2020. Relevant data were collected using electronic patient records and questionnaire-based, long-term, telephonic follow-up (median follow-up of 3.7 years). Outcomes examined were immediate/short-term complications (biliary injury, bile leak, return-to-theatre) and long-term problems (recurrent symptoms, choledocholithiasis, cholangitis/pancreatitis).

Results

There were a total of 50 STC cases (58% females) out of 4,341 cholecystectomies performed (1.15%), with the median age, body mass index, and length of stay being 69.5 years, 29 kg/m^2^ and eight days, respectively. Twenty-eight (56%) were emergency. No patient endured bile duct injury. Seven (14%) patients had postoperative bile leak which was significantly more common when Hartmann’s pouch was left open (33% vs. 8%; p = 0.03). No bile duct injury was reported. Most were managed conservatively (endoscopic retrograde cholangiopancreatography + stent: four; radiological drainage: one; no intervention: one). Only one patient required laparoscopic lavage and drainage. The true incidence of developing choledocholithiasis over the long term was 4/50 (8%) in our study. The median interval between STC and the diagnosis of postoperative choledocholithiasis was 15.9 months. All four patients had undergone type 1 STC (where the remnant of Hartmann’s pouch was closed with sutures); however, subsequent cross-sectional imaging (magnetic resonance cholangiopancreatography or computed tomography) showed that the gallbladder remnant was visible in only two of these four patients.

Conclusions

STC is a safe option in difficult situations and prevents bile duct injury. Although the risk of bile leak can be reduced by closing Hartmann’s pouch remnant, this may slightly increase the risk of subsequent stone formation. Infrequent occurrence of recurrent gallstone-related symptoms or complications favours its use.

## Introduction

Gallstone disease (GSD) is one of the most common general surgical presentations both in elective and emergency settings. Approximately 15% of the adult population in the United Kingdom (UK) is thought to have gallstones, with the majority of them being asymptomatic. The National Institute of Health and Care Excellence (NICE) in the UK recommends reassuring asymptomatic patients unless they develop symptoms [1]. For symptomatic or complicated GSD, cholecystectomy is the gold-standard treatment worldwide, with approximately 1-4% of people with gallstones undergoing this operation [2]. In England alone, approximately 70,000 cholecystectomies are performed every year [3]. In the emergency setting, GSD accounts for one-third of general surgery referrals and/or admissions [3,4]. In line with this, emergency cholecystectomy for complicated GSD (crescendo biliary colic, calculous cholecystitis, or pancreatitis) is also being performed more frequently in the UK as well as globally. Emergency cholecystectomy is recommended as soon as feasible and up to 10 days after an acute presentation due to gallstones by the World Society of Emergency Surgery [5] and within seven days of admission with acute cholecystitis by NICE [1].

Since the advent of laparoscopic surgery in the early 1990s, laparoscopic cholecystectomy (LC) has become the standard procedure for gallbladder removal throughout the world. Despite having a decent safety profile, complications do occur during LC. Some of them, especially those involving injury to the biliary tree, can be quite detrimental and difficult to repair. Bile duct injury results in high morbidity and sometimes even mortality [6]. The majority of patients with bile duct injury report reduced long-term quality of life [7]. The incidence of bile duct injury was 0.2-0.4% in a large population-based cohort study in the UK and Ireland with no statistical difference between emergency, delayed, or elective cholecystectomy [8]. In an attempt to avoid such injuries, one of the key principles of performing a safe LC is achieving a critical view of safety before dividing any structures. However, it is not always possible to achieve this critical view due to extensive scarring and a frozen hepatocystic triangle secondary to recurrent attacks of biliary sepsis. To deal with such difficult scenarios, several strategies have been described to safely bail out of challenging cholecystectomy, including the fundus-first approach, subtotal cholecystectomy (STC), and cholecystostomy [9]. STC involves opening Hartmann’s pouch to extract stones, removing the distal gallbladder, and either closing the proximal Hartmann’s pouch remnant or leaving it open. Traditionally, it has been classified as either *reconstituting* if the end of the remnant gallbladder is closed or *fenestrating* if the gallbladder remnant is left open but the cystic duct opening may be sutured internally [10]. More recently, Purzner et al. have proposed a more detailed classification for STC [11].

Despite STC being a well-documented, safe, and effective bail-out strategy, persistent symptoms of GSD are known to occur in 2.2% of patients according to a recent systematic review [12]. Moreover, evidence on the long-term outcomes of STC from the UK is scarce. Therefore, in this observational study with a retrospective as well as prospective component examining long-term follow-up, our main objectives were to investigate the early-to-medium-term morbidity associated with STC, such as biliary injury, bile leak, infection, reintervention and readmission rates, as well as the long-term sequelae of this surgical approach in terms of recurrent biliary events and the need for a completion cholecystectomy. We also sought to compare the outcomes of different types of STC, especially when Hartmann’s pouch is closed compared to being left open.

## Materials and methods

Study design and subjects

We conducted a retrospective analysis of all elective and emergency STCs performed in a single UK NHS Trust between 2014 and 2020. Patients were identified by searching the electronically recorded operation notes saved on the Trust’s hard drives using phrases such as “subtotal/sub-total/partial cholecystectomy.” Patients of all ages were included. Cholecystectomies performed as part of another operation were excluded. Ethical approval was obtained by registering the study with the Research and Audit Department of the Shrewsbury and Telford Hospitals NHS Trust, England, UK (approval number: 4818).

Data collection

Electronic patient records were used to collect data for each patient focusing on demographic characteristics and information about inpatient perioperative and short-to-medium-term postoperative period. In particular, we reviewed the results of laboratory tests, imaging and endoscopic investigations, operation notes, and discharge and subsequent clinic letters (if available) to gather relevant data on preoperative diagnosis, operative findings, intra- and/or postoperative complications, length of stay, and any recurrent presentations with suspected ongoing biliary problems.

To gather long-term outcome data, electronic patient records were again consulted to gather relevant postoperative information about recurrent admissions, investigations, or interventions. Furthermore, to collect rigorous long-term data, patients were followed up via telephone at least one year after their index operation using a standardised questionnaire (Table 1). Informed verbal consent was obtained before this. This questionnaire-based follow-up was meant to supplement the data gathered via electronic patient records and to fill any gaps in the data. As this was not meant to measure postoperative quality of life or patient-reported outcome measures, formal validation of the questionnaire was not required. Long-term outcomes data included any persistent or recurrent gallstone-related problems (e.g., biliary colic, choledocholithiasis, cholangitis, or pancreatitis) including assessment of frequency and severity of these problems and whether they led to further admissions, investigations, or interventions.

**Table 1:** Questionnaire used for collecting long-term follow-up data.

Questions
Q1. Overall, do you think that your gallbladder removal operation was successful in relieving you of the problems/symptoms you were experiencing due to gallstones? (Yes – fully) (Yes – partially) (Not sure) (No)
Q2. Since your initial gallbladder removal operation, have you suffered from any symptoms similar to what you had before the operation?
Q3. If yes to the above, what is the frequency and severity of these symptoms? (Mild/Moderate/Severe) (Daily/Weekly/Monthly/Less frequently than monthly)
Q4. Since your initial gallbladder removal operation, have you suffered from symptoms severe enough to require visiting or getting admitted to the hospital?
Q5. Since your initial gallbladder removal operation, have you ever been diagnosed with a gallstones-related complication such as jaundice, pancreatitis, or cholangitis (infection with jaundice)?
Q6. Since your initial gallbladder removal operation, have you undergone any endoscopic procedures to treat gallstone-related problems? For example, an endoscopic retrograde cholangiopancreatography (ERCP) to remove gallstones from the bile duct.
Q7. Since your initial gallbladder removal operation, have you undergone any further operation under general anaesthesia on your remaining gallbladder or bile duct?

Types of subtotal cholecystectomy

We used the classification of STC based on a previously published system by Purzner et al. [10]. Depending on whether Hartmann’s pouch is closed or the posterior wall is removed, STC has been classified as 1A (gallstones are removed; Hartmann’s pouch is closed and the posterior wall of the gallbladder is fully dissected off the liver bed), 1B (gallstones are removed; Hartmann’s pouch is closed and the posterior wall of the gallbladder is left in situ), 2A (gallstones are removed; Hartmann’s pouch is left open and the posterior wall of the gallbladder is fully dissected off the liver bed), 2B (gallstones are removed; Hartmann’s pouch is left open and the posterior wall of the gallbladder is left in situ), and type 3 (the fundus of the gallbladder is removed; gallstones are evacuated and the gallbladder remnant is left open).

Statistical analysis

Data were summarised using descriptive statistics. Non-continuous nominal data sets such as sex, American Society of Anesthesiologists (ASA) grade, STC type, complication, morbidity, and readmission rates were expressed as percentages, and, where applicable, were compared using 2 × 2 contingency tables and Fisher’s exact test. The Shapiro-Wilk test for normality determined that continuous variables (age, body mass index (BMI), length of stay, etc.) were non-parametric; therefore, continuous data were expressed as median and range, and, where applicable, were compared using a Mann-Whitney test. All statistical analyses were conducted using GraphPad Prism software, version 6 (San Diego, CA, USA). P-values <0.05 were considered statistically significant.

## Results

Patient demographics

Between 2014 and 2020, a total of 4,341 cholecystectomies were performed, of which 50 were STCs (1.15%). The median age and BMI of the patients were 69.5 years and 29 kg/m^2^, respectively. Female patients comprised 58% of the cohort (N = 29). The median overall length of stay (LOS) was eight days (range = 1-44 days). For emergency and elective cases, the median LOS was nine days (range = 3-25 days) and three days (range = 1-44 days), respectively (p = 0.03). The majority of the patients had ASA Grade 2 (N = 41, 82%). Specific comorbidities and their relative prevalence are detailed in Table 2. Seven patients had been treated for cancers in the past. Of these, there were two prostate cancer patients, three colorectal cancer patients, one uterine cancer patient, and one oral cancer patient.

**Table 2:** Demographics of the study participants. §: Median (range) used for non-parametric continuous data. ASA: American Society of Anesthesiologists; BMI: body mass index

Demographics	Number of patients (N) or Median (range)	%
Total number of patients	50	-
Age (years)	69.5 (27–87)^§^	-
BMI (kg/m^2^)	29 (24–43)^§^	-
Sex – Female	29	58%
Sex – Male	21	42%
Length of stay (days)	8 (1–44)^§^	-
ASA Grade		
ASA-1	2	4%
ASA-2	41	82%
ASA-3	7	14%
ASA-4	0	0%
Preoperative comorbidities		
Obesity (BMI ≥30 kg/m^2^)	19	38%
Hypertension	18	36%
Diabetes mellitus	8	16%
Ischaemic heart disease	8	16%
Myocardial infarction	6	12%
Atrial fibrillation	6	12%
Congestive cardiac failure	1	2%
Cerebrovascular disease	5	10%
Chronic pulmonary disease	9	18%
Chronic kidney disease	2	4%
Chronic liver disease	2	4%
Malignancy	7	14%
Peptic ulcer disease	3	6%
Connective tissue disease	6	12%
Venous thromboembolism	3	6%
Dementia	1	2%

Procedure details

Over the study period, the total number of cholecystectomies performed was 4,341, of which 730 were performed as an emergency. There were 50 cases of STC, 28 (56%) of which were performed as an emergency whereas 22 (44%) were elective. This gives an overall rate of STC of 1.15% (50 out of 4,341). The rates of STC being performed in the emergency and elective settings were 3.8% (28 out of 730 emergency cases) and 0.61% (22 out of 3,611 elective cases), respectively; this difference was statistically significant (relative risk = 6.29, 95% confidence interval (CI) = 3.62 to 10.94, p < 0.0001). All main operating surgeons were either consultants or associate specialists with extensive experience in laparoscopic surgery. All operations started via the laparoscopic approach but 10 had to be converted to open (20%) due to unfavourable anatomy and failure to progress safely through laparoscopy. The rate of conversion to open was 6/28 (21.4%) in emergency and 4/22 (18.2%) in elective cases, with no significant difference between the two (RR = 1.18, 95% CI = 0.38 to 3.67, p = 0.77). Dense adhesions and a contracted scarred gallbladder were responsible for open conversion in half of the cases (five out of 10). For the remaining, Mirizzi’s syndrome accounted for two and fistulae (cholecystoduodenal or cholecystocolonic) for four out of 10 conversions to open (Table 3). Mirizzi’s syndrome and a cholecystoduodenal fistula co-existed in one patient.

**Table 3:** Procedure details STC: subtotal cholecystectomy

Variable	Number of patients (N)	%
Mode of admission		
Emergency	28	56%
Elective	22	44%
Operative approach		
Laparoscopic	40	80%
Laparoscopic-to-open	10	20%
Operative findings		
Dense/extensive adhesions	24	48%
Frozen cystohepatic triangle	16	32%
Gangrenous gallbladder	12	24%
Perforated gallbladder	10	20%
Empyema	13	26%
Inflammatory phlegmon	10	20%
Biliary peritonitis	1	2%
Mirizzi’s syndrome	2	4%
Biliary fistulae	4	8%
Type of STC		
1A	32	64%
1B	6	12%
2A	5	10%
2B	6	12%
3	1	2%
Hartmann’s pouch closure technique for Type 1 STC (out of 38)		
Polyglactin – continuous	18	47.4%
Polyglactin – purse-string	5	13.1%
Polyglactin loop	2	5.3%
Polydioxanone – continuous	4	10.5%
V-loc™ (absorbable)	5	13.1%
Laparoscopic stapler	2	5.3%
Not known	2	5.3%
Secondary procedures		
Operative cholangiogram	3	6%
Bile duct exploration	3	6%
Repair of biliary fistulae	4	8%
Oesophagogastroduodenoscopy	1	2%
Parastomal hernia repair	1	2%

According to the Toronto classification of STC [10], 38 (76%) patients underwent Type 1 STC where Hartmann’s pouch was closed. The posterior gallbladder wall was dissected off the liver in 32 of these 38 cases (Type 1A, 64%). Continuous suture closure was the most common method of Hartmann’s pouch closure used in 27/38 cases (71%). The various suture materials and other methods employed for such closure are detailed in Table 3. Of the remaining 12 cases, 11 were Type 2 (22%) with a single case of Type 3 STC where only the fundus was opened to remove the stones with a drain being placed inside the gallbladder.

Drains were placed in the majority of cases, with the subhepatic space being the most common site for drain placement (n = 47, 94%). In two patients undergoing Type 2 STC, drains were left inside the open Hartmann’s pouch. Secondary procedures included operative cholangiogram in three (6%) cases and common bile duct (CBD) exploration in three (6%) cases. One patient underwent laparoscopic transcystic CBD exploration while the other two required open approach, both undergoing a T-tube drainage in the end. Repair of biliary fistulae was performed in four (8%) cases (one cholecystocolonic and three cholecystoduodenal), all requiring conversion to open.

Early postoperative and short-term complications

Complications of STC, either encountered immediately intraoperatively or in the short-term, are detailed in Table 4. None of the patients suffered a biliary injury. One patient suffered from a significant postoperative haemorrhage requiring re-laparoscopy and bleeding control from the liver bed. Six patients had unplanned re-presentation to the department within 90 days of their cholecystectomy. The median time to re-presentation was 14 days (range = 3-56 days). Two patients were re-admitted due to postoperative collections requiring radiological drainage. In one patient, the collection was due to a bile leak that also required an endoscopic retrograde cholangiopancreatography (ERCP) to control it.

**Table 4:** Early and short-term postoperative complications. ERCP: endoscopic retrograde cholangiopancreatography; ITU: intensive care unit; HDU: high dependency unit

Complications	Number of patients (N)	%	Clavien-Dindo (CD) grade
Bile duct injury	0	0	–
Bile leak (see Table 6 for details)	7	14%	CD-2: 1; CD-3a: 5; CD-3b: 1
Haemorrhage	1	2%	CD-3b (laparoscopy and haemostasis of liver bed bleed)
Intraabdominal collection	2	4%	CD-3a (radiological drainage)
Surgical site infection	3	4%	CD-2
Acute kidney injury	5	10%	CD-1
Anaemia	2	4%	CD-2
ITU/HDU admission	1	2%	CD-4b
90-day mortality	1	2%	–
90-day re-presentation	6	12%	–
Reasons for re-presentation			
Intraabdominal collection	2	–	–
Ongoing abdominal pain	2	–	–
Post-ERCP mild pancreatitis	1	–	–
Wound infection	1	–	–

Postoperative bile leak

Postoperative bile leak was seen in seven patients (Table 5). Bile leak patients had a significantly longer median length of stay (13 vs. 8 days; p = 0.03) and a 90-day re-presentation rate (42.8% vs. 7%; p = 0.007). The rate of bile leak was significantly higher in patients undergoing Type 2/3 STC. Four out of 12 (33.3%) patients who underwent a Type 2/3 STC suffered from a postoperative bile leak compared to only three out of 38 (7.9%) Type 1 STC patients (odds ratio (OR) = 5.8, 95% CI = 1.084 to 31.39, p = 0.04). Emergency compared to elective presentation did not have any effect on the risk of bile leak (14.3% emergency, 13.6% elective; p = 1.00). The median time to intervention was five days (range = 2-12 days). None of the patients required escalation to level 2 or 3 care and there was no mortality.

**Table 5:** Postoperative bile leak *: Fisher’s exact test used to calculate statistical significance. BMI: body mass index

Variable	Bile leak (N = 7)	No bile leak (N = 43)	P-value*
Median age in years (range)	60 (50–87)	70 (27–84)	0.21
Median BMI (kg/m^2^)	33	29	0.27
Median length of stay in days (range)	13 (5–25)	8 (1–44)	0.03
90-day re-presentation rate (%)	3/7 (42.8%)	3/43 (7%)	0.007
Gallbladder remnant left open	4/12 (33.3%)	8/12 (66.7%)	0.04
Gallbladder remnant closed	3/38 (7.9%)	35/38 (92.1%)	

Individual characteristics and management of bile leaks in each of these patients are summarised in Table 6. Four patients were managed by ERCP and stenting, one required computed tomography (CT)-guided drainage, and one patient with biliary peritonitis underwent a re-laparoscopy, washout, and further drain placement. In one patient, the bile leak slowly stopped without any intervention. Most bile leak patients made uneventful recoveries with no long-term sequelae except one patient who presented more than a year later with choledocholithiasis and cholangitis.

**Table 6:** Characteristics and management of patients with postoperative bile leak. ASA: American Society of Anesthesiologists; ERCP: endoscopic retrograde cholangiopancreatography

Patient	Age/Sex	ASA	Preoperative ERCP	Mode of admission	Technique of Hartmann’s pouch closure	Management	Timing of postoperative intervention (days postoperatively)	Length of stay (days)
1	58 M	2	No	Emergency	V-loc™ (absorbable)	ERCP + stent	8	7
2	87 M	3	No	Emergency	V-loc™ (absorbable)	ERCP + stent	12	21
3	50 F	2	No	Elective	Polyglactin – purse-string	Laparoscopy and washout for biliary peritonitis. The purse-string suture had cut through making the ligature loose. The leak site closed with two interrupted sutures.	2	13
4	70 M	3	No	Elective	NA	ERCP + stent	2	5
5	50 F	2	No	Elective	NA (drain left in Hartmann’s pouch)	ERCP + stent	8	14
6	60 M	2	Yes	Emergency	NA	Bile leak sealed spontaneously	NA	13
7	60 F	3	No	Emergency	NA	Bile leak secondary to duodenal perforation. Managed by CT-guided drainage and antibiotics	2	25

Incidence of choledocholithiasis

The total number of patients who had choledocholithiasis at any time (pre- and postoperatively) was 17 (34%) (Table 7). Eleven (22%) patients had a preoperative diagnosis of ductal stones, with nine (18%) having successful ERCP and CBD clearance in the past. Two (4%) patients had known choledocholithiasis with CBD stents at the time of STC as their preoperative ERCP failed to clear the CBD; however, postoperatively, both of them had successful ERCP and CBD clearance. Two patients were diagnosed with choledocholithiasis either intraoperatively (73, female) or in the early postoperative period (50, female). Only four (8%) patients suffered from ductal stones in the long term which may represent the true incidence of postoperative choledocholithiasis in our study. The median interval between STC and diagnosis of choledocholithiasis was 15.9 months. All four patients had undergone Type 1 STC (where the remnant of Hartmann’s pouch was closed with sutures). Only one of these four patients (87, male) also suffered from a bile leak in the immediate postoperative period which was treated with ERCP and stenting. Subsequent cross-sectional imaging (magnetic resonance cholangiopancreatography and/or CT) had shown that the gallbladder remnant was visible in only two of these four patients but no obvious stones were seen in these two remnant gallbladders at the time of imaging. Therefore, it is not clear if the postoperative choledocholithiasis was the result of stone migration from the remnant gallbladder or de novo CBD stone formation.

**Table 7:** Incidence of choledocholithiasis. CBD: common bile duct; ERCP: endoscopic retrograde cholangiopancreatography; GB: gallbladder; STC: subtotal cholecystectomy

Patient	Age/Sex	ASA	Interval between STC and the diagnosis of choledocholithiasis	Type of STC	Is GB remnant visible on postoperative imaging?	Postoperative bile leak?	Management
Preoperative or early postoperative diagnosis							
1	50 F	2	N/A (choledocholithiasis diagnosed in the early postoperative period)	1A	No imaging	Yes	ERCP and successful duct clearance (NB: also suffered from postoperative bile leak and biliary peritonitis, requiring re-laparoscopy and washout)
2	73 F	3	N/A (choledocholithiasis diagnosed at the time of STC)	2B	No	No	CBD exploration performed at the time of STC but unsuccessful in clearing the duct. ERCP (postoperative) and successful duct clearance
3	66 F	2	N/A (choledocholithiasis confirmed and CBD stented prior to the operation)	1B	No imaging	No	ERCP, stent removal and successful duct clearance (NB: had biliary peritonitis due to perforated gangrenous GB at the time of index operation)
4	68 F	2	N/A (choledocholithiasis confirmed and CBD stented prior to the operation)	1A	No GB remnant but CBD stone	No	Repeat ERCP unsuccessful, hence stent replaced. Pt underwent open CBD exploration and stone removal; CBD closed over a T-tube
Late postoperative diagnosis (true choledocholithiasis in the long term)							
5	87 M	3	13 months	1B	Yes, no stones seen	Yes	Died of biliary sepsis
6	67 F	2	26.5 months	1A	Yes, with CBD stone	No	ERCP and successful duct clearance
7	79 M	2	16.5 months	1A	No GB remnant but CBD stone	No	ERCP and successful duct clearance
8	27 F	2	15.3 months	1A	No GB remnant but CBD stone	No	Recurrent “de novo” CBD stone formation with 2× successful ERCP. Finally, referred to a tertiary HPB centre for consideration of biliary reconstruction

Long-term follow-up

Long-term telephonic follow-up was conducted using the questionnaire presented in Table 1. It focused on the long-term sequelae of STC in terms of recurrent or persistent symptoms, gallstone-related complications (choledocholithiasis, cholangitis, or pancreatitis), and the need for any further endoscopic or surgical interventions. Information about long-term follow-up could be obtained from about 37 patients (74% follow-up rate). The median duration of follow-up was 44.6 months (range = 9.6-91 months). The results of the long-term follow-up are summarised in Table 8. Eight out of the 37 patients presented again with symptoms suspected or proven to be due to gallstones. The median time to re-presentation was 15 months (range = 4.4-26.5 months) and the most common reason for re-presentation was choledocholithiasis (five out of eight patients); four of these patients also had concurrent cholangitis and went on to have ERCP which was successful in clearing the bile duct in three; one patient had two unsuccessful ERCPs and finally required an open CBD exploration and duct clearance. One cholangitis patient (87, male) could not have ERCP due to severe sepsis and died as a result of it. This accounted for one of the two long-term deaths in this cohort, the second one being due to unrelated causes several years after the index cholecystectomy. All patients who presented several months later with choledocholithiasis had undergone Type 1 STC at the time of their index operation. One patient who suffered daily severe symptoms and was re-admitted once had undergone a Type 2 STC. Completion cholecystectomy was required in two patients and both of them had undergone a Type 1 (re-constituting) STC previously (one laparoscopic and one open). Re-do operations were technically challenging but both were completed laparoscopically.

**Table 8:** Long-term follow-up. CBD: common bile duct; ERCP: endoscopic retrograde cholangiopancreatography

Question	Number of patients (out of 37)	%
Is STC successful in relieving symptoms?		
Yes – fully	26	70.3%
Yes – partially	9	34.3%
No	2	5.4%
Are recurrent symptoms severe enough to require hospital visit or admission?		
Total	7	18.9%
Re-admitted	5 (4 with cholangitis)	13.5%
Visited only	2	5.4%
Postoperative gallstone complications?		
Pancreatitis	0	-
Choledocholithiasis	1	2.7%
Choledocholithiasis with cholangitis	4	10.8%
Further procedure(s)?		
Endoscopic (ERCP)	4	10.8%
CBD exploration (open)	1	2.7%
Completion cholecystectomy	2	5.4%

## Discussion

The severity of acute cholecystitis can be classified according to intraoperative findings and organ dysfunction per the 2012 Tokyo guidelines [12]. Patients with Tokyo Grade II or higher were found to be at a higher risk of morbidity from bile duct injuries due to extensive inflammation and fibrosis in the hepatocystic triangle [13]. Different damage control surgery techniques have been described in the literature to deal with this situation. One of these strategies is STC which involves opening the gallbladder anteriorly, followed by stone extraction and removal of part of the gallbladder. STC was first suggested as an alternative approach in difficult cases by Madding in 1955 [14]. Bickel and Shtamler published their eight-year experience of laparoscopic STC in 1993 with no iatrogenic bile duct injury [15]. In a recent Delphi consensus on bile duct injuries, STC was favoured by the majority of participants as an approach to minimise bile duct injury risk [6].

STC has been described as a strategy to avoid adversity (bile duct injury). The Tokyo guidelines [12] suggest a relationship between the severity of acute cholecystitis and the incidence of bile duct injury. However, we found no statistically significant difference in the frequency of STC when it comes to emergency or elective surgery. This suggests that chronic cholecystitis can pose challenges to safe dissection in Calot’s triangle as much as acute severe cholecystitis. Our conversion rate (laparoscopic to open) was similar to a meta-analysis published in 2015 [16].

In terms of immediate postoperative complications, an overall bile leak rate of 14% (8% for Type 1 and 33% for Type 2 STC) in our study was comparable to the reported incidence of 13.9% in a recent meta-analysis [17]. In our practice, closure of Hartmann’s pouch was the more frequent approach (Type 1 or reconstituting STC), which is similar to the study by Purzner et al. [10], describing these types in detail. As expected, this significantly reduces the risk of postoperative bile leak. Strasberg et al. suggested that the fenestrating type (closure of cystic duct with purse-string suture) was a better approach in comparison to the reconstituting type [9]. However, this argument has been negated by a large cohort study [18] and a recently published meta-analysis [17] favouring the reconstituting approach over the fenestrating approach supporting our department’s preference. Although the bile leak did prolong the hospital stay, it was manageable in most cases through non-operative means and did not lead to any untoward long-term sequelae.

The rate of postoperative choledocholithiasis after Type 1 STC was 10.52% (four out of 38). It is unclear whether these were retained or de novo stones but looking at the median interval between STC and choledocholithiasis, de novo stone formation appears more plausible explanation. Examining the long-term data, it appears that the reconstituting (Type 1 STC) approach is associated with the risk of subsequent choledocholithiasis or other GSD-related symptoms because, in principle, it creates a “neo” gallbladder by closing Hartmann’s pouch. The scale of this problem would also depend on the size of the gallbladder remnant left behind.

It is important to note that recurrent attacks of cholecystitis or cholangitis as well as long waiting times between the onset of symptomatic GSD and definitive surgical intervention lead to extensive scarring in the region of the Calot’s triangle. This makes dissection challenging, forcing surgeons to opt for a bail-out strategy at times in the form of STC. Although STC is a safe procedure for preventing injury to the bile duct, it does have its own problems and limitations. For instance, bile leak in the short term and incidence of further stone formation in the gallbladder remnant in the longer term. We believe that the best approach to avoid these problems is to minimise the interval between the onset of symptoms and surgical intervention thus preventing recurrent attacks and to increase the provision of emergency cholecystectomy within the safe window of about four to five days from the onset of acute cholecystitis.

There are limitations to our study. We have examined long-term outcomes of STC with a median follow-up of 44.6 months with a direct comparison of Type 1 and Type 2 STC. However, short- and medium-term data collection was retrospective. Furthermore, a significantly larger number of STC cases would need to be looked at to properly investigate the true risk of biliary injury during STC as the rate of this complication even for a total cholecystectomy is only 0.2-0.33%. Similarly, the numbers of Type 2 STC are much smaller and higher numbers would be required for a better comparison between the outcomes of different STC types. We acknowledge that this may not be easy to achieve due to surgeons’ tendency to close Hartmann’s pouch.

## Conclusions

This study supports the practice of laparoscopic STC as a pragmatic option when safe dissection in the hepatocystic triangle is difficult. It can minimise the risk of potentially devastating bile duct injury while allowing the surgeon to remove most of the gallbladder. The risk of conversion to open operation is also reduced, the latter being associated with considerably higher morbidity and length of stay. Type 1 or reconstituting type of STC seems to be the more popular choice among surgeons as it reduces the risk of postoperative bile leak. However, most bile leaks can be managed with conservative or non-operative management without any untoward long-term consequences. On the other hand, closing the pouch and reconstituting a “neo” gallbladder in our study showed a significantly higher incidence of long-term issues such as recurrent biliary colic and choledocholithiasis. Larger studies with proportionally equal numbers of different types of STC would be required to investigate the differences in the long-term outcomes of each type.
